# Silencing *ACE1* Gene with dsRNA of Different Lengths Impairs Larval Development in *Leptinotarsa decemlineata*

**DOI:** 10.3390/insects15121000

**Published:** 2024-12-17

**Authors:** Brenda Julian-Chávez, Tania S. Siqueiros-Cendón, Jorge Ariel Torres-Castillo, Sugey Ramona Sinagawa-García, María Jazmín Abraham-Juárez, Carmen Daniela González-Barriga, Quintín Rascón-Cruz, Luis Ignacio Siañez-Estrada, Sigifredo Arévalo-Gallegos, Edward Alexander Espinoza-Sánchez

**Affiliations:** 1 Laboratorio de Biotecnología I, Facultad de Ciencias Químicas, Universidad Autónoma de Chihuahua, Circuito Universitario S/N Nuevo Campus Universitario, Chihuahua 31125, Chihuahua, Mexico; p363961@uach.mx (B.J.-C.); tsiqueiros@uach.mx (T.S.S.-C.); qrascon@uach.mx (Q.R.-C.); lsianez@uach.mx (L.I.S.-E.); sareval@uach.mx (S.A.-G.); 2 Instituto de Ecología Aplicada, Universidad Autónoma de Tamaulipas, Ave. División del Golfo 356, Col. Libertad, Ciudad Victoria 87019, Tamaulipas, Mexico; joatorres@docentes.uat.edu.mx; 3 Laboratorio de Biotecnología, Facultad de Agronomía, Universidad Autónoma de Nuevo León, Francisco Villa S/N Col. Ex hacienda El Canadá, General Escobedo 66050, Nuevo León, Mexico; sugey.sinagawagr@uanl.edu.mx; 4 Centro de Investigación y de Estudios Avanzados del IPN, Libramiento Norte León Km 9.6, Irapuato 36821, Guanajuato, Mexico; jazmin.abraham@cinvestav.mx; 5 Laboratorio de Cultivo de Tejidos, División de Ingeniería y Ciencias, Tecnológico de Monterrey, Av. Heroico Colegio Militar 4700, Nombre de Dios, Chihuahua 31100, Chihuahua, Mexico; cgonzalezb@tec.mx

**Keywords:** *Leptinotarsa decemlineata*, acetylcholinesterase, *ACE1* gene, RNA interference, dsRNA length, transcript levels, mortality

## Abstract

In the search for effective strategies to control the Colorado Potato Beetle, RNA interference technology has emerged as a promising method due to its capacity to suppress genes selectively. Factors such as the target gene and double-stranded RNA (dsRNA) length are critical for optimizing gene silencing efficiency. In this study, we designed and synthesized in vitro dsRNAs of varying lengths targeting the *ACE1* gene, which encodes the AChE1 isoform of acetylcholinesterase in the beetle. All tested dsRNA lengths (222 bp, 543 bp, 670 bp, and 870 bp) promoted transcript reduction. The 670 bp dsRNA was the most effective, reducing transcript levels by approximately 40% by day seven, followed by the 543 bp dsRNA. No significant differences were observed between the 222 bp and 870 bp dsRNAs. Furthermore, all of the dsRNA lengths resulted in reduced weight gain and increased mortality in larvae, with the 670 bp dsRNA showing the highest mortality rate, leaving only 63% larval survival, a trend that persisted through day nine. These findings emphasize that dsRNA length is a key factor in the silencing response, underscoring the importance of selecting the optimal length while considering the gene’s target, stability, and delivery methods. This study contributes to establishing design criteria for dsRNA, aiding in the development of more effective and sustainable pest management strategies.

## Simple Summary

The Colorado Potato Beetle has become a super pest capable of resisting various insecticides. In the search for effective alternatives to control it, RNA interference has emerged as a promising technology to silence essential genes. However, its effectiveness can be influenced by several factors. One critical factor is determining the optimal length of double-stranded RNA required to achieve a strong silencing effect on the beetle’s genes. We designed double-stranded RNAs of different lengths targeting the *ACE1* gene of the beetle. Our findings revealed that the dsRNA must have a length and stability to remain intact until it reaches the target cells. This research provides valuable insights to enhance the efficiency of gene-silencing strategies, contributing to the development of sustainable pest control methods in agriculture.

## 1. Introduction

The Colorado Potato Beetle (CPB, *Leptinotarsa decemlineata* Say, Coleoptera: Chrysomelidae) is considered the most serious pest affecting potato crops worldwide, causing severe damage through defoliation by both larvae and adult beetles. This impact leads to significant yield losses, with estimates of economic damage reaching USD 2–2.5 billion annually in countries like Russia [1,2,3]. Over time, various strategies have been implemented to manage CPB populations, including cultural practices like crop rotation [4], biocontrol measures using *Beauveria bassiana* [5], and natural enemies such as *Chrysoperla carnea* and *Pterostichus melanarius* [6,7]. While these strategies offer varying levels of success, CPB continues to challenge agricultural resilience due to its adaptability.

Despite considerable efforts and investment in integrated pest management, chemical control remains the primary tool against CPB [8]. However, its rapid evolution regarding its resistance to insecticides is alarming [9]. According to the Arthropod Pesticide Resistance Database (APRD), by 2024, 427 cases of resistance to 57 active ingredients in CPB had been documented, with 121 occurring solely in 2024 [10].

In pursuit of sustainable alternatives, Cry proteins, known for their targeted action against pests, have been tested. While these proteins have shown some effectiveness against CPB [11,12,13], concerns persist regarding their potential impacts on non-target organisms [14,15]. Moreover, resistance to Cry proteins is already an issue: 26 cases involving Cry1Ab, Cry1Ac, Cry1A.105, Cry1Fa, Cry2Ab, Cry3Bb, mCry3A, eCry3.1Ab, and Cry34/35Ab have been reported across various pest species [16,17,18]. The constant exposure of CPB to Cry proteins heightens the risk of resistance developing [1,19].

Recently, RNA interference (RNAi) has been reported as a method to control various pests. This is a biological mechanism for post-transcriptional gene silencing activated by dsRNA, which, when processed by the RNase III enzyme Dicer (~200 kDa), produces 21–24 nucleotide fragments [20]. These fragments are then incorporated into the RNA-induced silencing complex (RISC), enabling targeted cleavage of complementary messenger RNA (mRNA) strands, thereby preventing their translation into proteins [21].

RNAi has been effectively implemented through various approaches, including the expression of dsRNA in bacteria, in vitro synthesis, the development of transgenic plants with nuclear-modified genomes, and, more recently, the integration of RNAi into the chloroplast genome [22,23,24]. These innovative approaches have shown promising results against a variety of agricultural pests, including *Diabrotica virgifera virgifera* [25], *Helicoverpa armigera* [26,27,28], *Myzus persicae* [29,30,31], *Bemisia tabaci* [32,33], *Sitobion avenae* [34], *Diaphorina citri* [35], *Plutella xylostella* [36], and *Chilio suppressalis* [37,38]. Notably, RNAi has proven to be highly specific, with a minimal impact on non-target organisms, facilitating the development of RNAi-based products now available on the market, such as the maize variety SmartStax PRO^®^ [39,40,41] and Calantha^TM^ (Ledprona) [42,43].

RNAi has also been tested against *L. decemlineata,* effectively silencing more than 26 genes, which has led to a decrease in larval weight, reduced growth, arrest of molting, alterations in amino acid metabolism, flight impairment, and mortality (Table 1).

Despite the significant advancements in RNAi technology, several aspects of RNAi sequence structures remain poorly understood. For instance, sequences that exhibit excessive structure or are rich in specific nucleotide regions may hinder efficient binding to the RISC, thereby impairing the effectiveness of RNAi [65]. Additionally, careful selection of target sites within the mRNA is crucial, as the efficacy of RNAi is highly contingent on the chosen binding location. It has been suggested that targeting intronic regions, as well as the 5′ untranslated region (UTR) and 3′ UTR, should be avoided [66]. Furthermore, it has been proposed that regions near the start codon should be approached with caution due to their high affinity for regulatory proteins; however, practical experience indicates that RNAi targeting these sites can yield effective outcomes [67].

To address the challenges associated with selecting target sites for RNAi, there has been growing emphasis on the design and production of long RNAs. This innovative approach enables the cellular machinery of insects to efficiently process extended dsRNA sequences, resulting in the generation of multiple small RNAs that enhance silencing efficacy. Although various studies have explored this methodology [23,24,68,69,70], uncertainty persists regarding the optimal length of long RNAi sequences required for effective gene silencing. Different RNAi lengths have been used in the literature, but no consensus has been reached on the most effective length. While it is generally accepted that sequences ≤ 60 bp are less effective than those of 200 bp [68,71], this issue remains a critical area for future research into RNAi and its application in pest control.

Therefore, we evaluated different dsRNA lengths targeting the *ACE1* gene, which encodes the AChE1 isoform of acetylcholinesterase in *L. decemlineata*. Silencing the *ACE1* gene via RNAi has been shown to impair insect physiology and reduce fitness [28,72,73]. Our goal was to identify the dsRNA length that produces the highest level of gene silencing and maximizes larval damage. This work contributes to a deeper understanding of RNAi efficacy in pest management, particularly in optimizing the design of RNAi constructs for enhanced gene interference.

## 2. Materials and Methods

### 2.1. Selection of the Target Gene

Acetylcholinesterase (AChE) is a critical enzyme that terminates neurotransmission and is a crucial target for anticholinesterase insecticides; therefore, it was selected for this study. The *ACE* sequences from *L. decemlineata* were retrieved from the National Center for Biotechnology Information (NCBI) database. To investigate the relationship between sequences, both DNA and amino acid alignments were performed using EMBOSS Water (https://www.ebi.ac.uk/jdispatcher/psa/emboss_water (accessed on 13 August 2024)). Structural modeling was conducted using Swiss-Model (https://swissmodel.expasy.org/ (accessed on 18 August 2024)), and structural comparisons were carried out via the DALI Protein Structure Comparison Server (http://ekhidna2.biocenter.helsinki.fi/dali/ (accessed on 19 August 2024)) [74]. BLAST analysis was used to identify sequences related to *ACE* genes.

To determine the most suitable target gene, we analyzed the binding affinities of the AChE1 and AChE2 isoforms to the acetylcholinesterase inhibitors, malathion (DrugBank: DB00772), and physostigmine (PubChem: 5983) through molecular docking analysis using AutoDock Vina [75], with an exhaustiveness setting of 100.

### 2.2. In Silico Analysis of RNAi Effectiveness and Off-Targets

Fragments of different lengths from the *ACE* gene were analyzed for their theoretical RNA interference effectiveness and potential off-target effects using the QUT *Nicotiana benthamiana* RNA Target Tester (https://benthgenome.qut.edu.au/tools/TKsoft3x.php (accessed on 11 November 2024)). The analysis was performed against *ACE* gene sequences from *Phyllotreta striolata* (MW149303), *Tribolium castaneum* (HQ260968), *Apis mellifera* (KU532288), *B. tabaci* (EF675188), *P. xylostella* (AY970293), *C. suppressalis* (KP657634)*, Bombyx mori* (DQ186605)*, Melitaea cinxia* (GQ489250), *Caenorhabditis elegans* (NM_078259.9), *Mus musculus* (EU623420), *Oryctolagus cuniculus* (U05036)*, Bos taurus* (NM_001076220), and *Homo sapiens* (M55040).

### 2.3. Cloning and Synthesis of ACE Fragments

Acetylcholinesterase gene fragments were processed using SnapGene 5.4.3 software (https://www.snapgene.com (accessed on 20 September 2024)), adding *Swa*I and *Avr*II restriction sites to the 5′ and 3′ ends for cloning. To ensure that the observed effects of dsRNA were due to length, sequences were selected to have approximately 60% GC content. The final sequences were synthesized by GeneScript Inc. (Piscataway, NJ, USA) and cloned into the pBJC4 vector (PQ589839). Recombinant vectors were introduced into *Escherichia coli* strain DH5α (Invitrogen^®^, Carlsbad, CA, USA) for plasmid maintenance and propagation.

### 2.4. In Vitro dsRNA Synthesis

In vitro synthesis of dsRNA was performed using the commercial T7 RiboMAX™ Express RNAi System kit (Promega, cat. P1700, Madison, WI, USA), following the manufacturer’s instructions. Plasmid DNAs containing the four different RNA lengths were obtained from *Escherichia coli* DH5α using the QIAGEN Plasmid Midiprep Kit (QIAGEN^®^, Valencia, CA, USA). The plasmid DNA was then used to amplify templates for in vitro transcription. For each dsRNA length, sense and antisense amplifications were carried out using specific primers synthesized by Integrated DNA Technologies (IDT). The minimal *T7* promoter sequence (5′TAATACGACTCACTATAGG3′) was incorporated at the 5′ end of both the forward and reverse primers (Table 2). The amplifications were performed under the following conditions: 3 min of denaturation at 94 °C, followed by 40 cycles of amplification (45 s at 94 °C, 45 s at 61 °C, 1 min at 72 °C), and a final extension step of 5 min at 72 °C.

The transcription reaction was set up as follows: 10 µL of RiboMAX™ Express T7 buffer, 2 µL of T7 Express Enzyme Mix, and 200 ng of both sense and antisense DNA, which had been previously amplified, making a final volume of 20 µL. The reaction mixture was incubated at 37 °C for 1 h, followed by a heat treatment at 70 °C for 10 min. Afterward, the mixture was allowed to cool gradually to room temperature over 20 min to ensure proper alignment of the RNA strands. To eliminate single-stranded RNA (ssRNA) and template DNA, the reaction was treated with RNase A (Thermo Fisher Scientific, Waltham, MA, USA) and DNase I (Thermo Fisher Scientific, Waltham, MA, USA) by incubating at 37 °C for 30 min. Subsequently, dsRNA was precipitated using 3 M sodium acetate (pH 5.2) and isopropanol. The resulting pellet was washed with cold 70% ethanol and resuspended in nuclease-free water. The integrity of the dsRNA was assessed using electrophoresis on a 1.8% agarose gel, and its concentration was quantified by measuring absorbance at 260 nm using a UV/VIS NanoDrop One Spectrophotometer (Thermo Fisher Scientific, Waltham, MA, USA). dsRNA was stored at −70 °C until use.

### 2.5. Insect Growth Conditions

Adult beetles of *L. decemlineata* were collected in the field from silverleaf nightshade (*Solanum elaeagnifolium* Cav.) and buffalobur (*Solanum rostratum* Dunal) plants in Chihuahua, Chihuahua, Mexico (28°43′47″ N, 105°58′11″ W). The beetles were maintained in a terrarium under controlled conditions with a temperature of 26 ± 2 °C, relative humidity of 50–60%, and under a 14 h light–10 h dark photoperiod (1200 lux). Silverleaf nightshade plants were provided as food.

To standardize larval size for growth and survival assays, adult CPB were allowed to lay eggs, which were then collected and transferred to fresh wild silverleaf nightshade leaves to hatch. First-instar larvae were allowed to feed on young leaves from one-month-old silverleaf nightshade plants.

For RNAi feeding experiments with different dsRNA lengths, synchronized groups of second-instar larvae were selected, individually weighed, and divided into three groups, each containing 27 larvae, which served as a biological replicate. Prior to the assay, the larvae were deprived of food for 6 h. Subsequently, each group was placed in Petri dishes and fed 23 cm^2^ leaf disks of silverleaf nightshade, which were painted with dsRNA diluted in nuclease-free water to a final concentration of 4 ng cm^−2^ [68]. Silverleaf nightshade leaves painted with nuclease-free water were used as controls [68]. The leaves were replaced with fresh dsRNA-painted leaves every 24 h for 8 d. Larvae were weighed on 3, 5, 7, and 9 d of feeding on dsRNA-painted leaves, and mortality was recorded daily. The larvae were stored at −70 °C until further processing.

### 2.6. RNA Extraction

For RNA extraction, 50–100 mg of frozen larvae were homogenized in liquid nitrogen, followed by RNA extraction using the Total RNA Purification Kit (Jena Bioscience, Jena, Germany) according to the manufacturer’s protocol. The extracted RNA was subsequently treated with DNase (Thermo Fisher Scientific, Waltham, MA, USA) to remove any contaminating DNA. The integrity of the RNA was evaluated by electrophoresis on a 1% agarose gel. RNA concentration was quantified by measuring absorbance at 260 nm using a UV/VIS NanoDrop One Spectrophotometer (Thermo Fisher Scientific, Waltham, MA, USA).

### 2.7. Quantitative Real-Time PCR (qPCR)

qPCR was employed to evaluate the transcript levels of *ACE1* from larval tissues. Primers for qPCR analysis were designed using Primer3 (https://primer3.ut.ee (accessed on 18 July 2024)) and the RealTime qPCR tool from IDT (https://www.idtdna.com/scitools/ (accessed on 18 July 2024)) and further analyzed with the OligoAnalyzer™ Tool (https://www.idtdna.com/calc/analyzer (accessed on 18 July 2024)). The specific primers used to amplify *ACE1* were Fw—5′TGACGACGATGATTCTGATTCC3′ and Rv—5′CCTGGTGAAGTCGCGTTT3′. Additionally, the primers Fw—5′GCGGGAGAATGTACAGAGGA3′ and Rv—5′AAGTCTTCACGGAGCTTGGA3′ were used to amplify the *Rps18* gene [24]. All primers were synthesized using IDT.

For cDNA synthesis, Super Script^®^ IV Reverse Transcriptase (Thermo Fisher Scientific, Waltham, MA, USA) was utilized, following the manufacturer’s guidelines. The reverse primers were aligned with the template RNA, and the reaction mixture was prepared as follows: 2 µM of gene-specific reverse primer, 10 mM dNTP mix (Promega), and 500 ng of RNA template in a final volume of 13 µL. The reaction mixture was incubated at 65 °C for 5 min, followed by cooling on ice for 1 min. The RT reaction was prepared by mixing 5X SSIV Buffer, 100 mM DTT, and 200 units of Super Script IV^®^ Reverse Transcriptase in a final volume of 7 µL. RT mixture was then added to the aligned RNA and incubated at 55 °C for 10 min, followed by 80 °C for 10 min to inactivate the reaction.

qPCR was performed using the following reaction mixture: 5 µL of iQ SYBR^®^ Green Supermix (Bio-Rad, Hercules, CA, USA), 5 µM of forward primer, 5 µM of reverse primer, and 2 µL of cDNA, resulting in a final reaction volume of 10 µL. The mixture was placed in a CFX96 Real-Time System (Bio-Rad, Hercules, CA, USA) and subjected to the following thermal cycling conditions: an initial denaturation step at 95 °C for 1 min, followed by 39 amplification cycles (denaturation at 95 °C for 10 s, annealing at 52 °C for 30 s, and extension at 65 °C for 5 s). The results were normalized to the mRNA levels of the *Rps18* gene, encoding ribosomal protein S18 in CPB [24], which served as a housekeeping gene. Relative expression levels of the *ACE1* gene were calculated using the ΔΔCt method [76]. Each reaction was repeated three times to minimize intra-experimental variation [24], and all results were analyzed using Bio-Rad CFX Manager 3.1 software.

### 2.8. Statistical Analysis

Data analysis was performed using Minitab^®^ 20.3 statistical software. A one-way ANOVA was conducted with a significance level set at *p* ≤ 0.05. Post hoc comparisons were made using Tukey’s mean separation test.

## 3. Results

### 3.1. Selection and Analysis of the ACE Gene for Silencing

Acetylcholinesterase is an essential enzyme for synaptic transmission in the central nervous system of *L. decemlineata* and, therefore, a target of two main insecticide families: organophosphates and carbamates. *L. decemlineata* has two genes, *ACE1* and *ACE2,* which encode AChE1 and AChE2 acetylcholinesterase isoforms.

To identify the appropriate *ACE* gene for silencing, we analyzed *ACE* sequences available in the NCBI database. We identified two sequences that encode acetylcholinesterase in *L. decemlineata*: *ACE1*, accession JF343436, and *ACE2*, accession JF343437, both reported by Revuelta et al. [77]. However, a third sequence described as *ACE* (accession L41180) by Zhu and Clark [78] was also recovered. Therefore, nucleotide and amino acid sequence alignments were conducted to discern whether L41180 corresponded to *ACE1*, *ACE2*, or an alternative variant.

The alignment analysis revealed that L41180 shared 50.9% nucleotide identity with *ACE1* and 99.2% with *ACE2*. The amino acid alignment showed 40.3% identity between L41180 and AChE1 and 99.7% identity with AChE2, differing by only two amino acid substitutions (Asp30Lys and Ser290Gly). These results indicate that *ACE* (L41180) and *ACE2* (JF343437) encode essentially the same protein. However, structural comparison analysis revealed a root mean square deviation (RMSD) of 0.8, suggesting slight variations that could impact their functional characteristics. We performed docking analyses with the acetylcholinesterase inhibitors malathion and physostigmine to investigate whether changes in these two amino acids might provide an evolutionary advantage. The results indicate that the AChE2 encoded by *ACE* (L41180) had affinities of −5.3 and −9.5 Kcal/mol, respectively, while the AChE2 encoded by *ACE2* (JF343437) showed affinities of −5.0 and −8.6 Kcal/mol. The reduced affinity of *ACE2* (JF343437) suggests that it is less sensitive to these inhibitors. Considering the adaptive mutation potential of *L. decemlineata* and given that organisms with advantageous evolutionary sequences tend to be conserved, we hypothesize that *ACE2* (JF343437), which provides this evolutionary advantage, is more prevalent in current CPB populations and was to be used as a reference sequence.

After determining that *ACE2* (JF343437) should be used as the reference gene sequence, we analyzed the protein isoforms it encodes. Molecular docking analysis was then performed on the AChE1 and AChE2 proteins—encoded by *ACE1* and *ACE2*, respectively—using physostigmine as the ligand. This analysis showed that AChE1 had a higher binding affinity for physostigmine (−9.0 Kcal/mol) compared to AChE2 (−8.6 Kcal/mol). These results suggest that AChE1 interacts more strongly with physostigmine than AChE2, which supports the hypothesis that acetylcholinesterase inhibitors exert their lethal effect in *L. decemlineata* by interacting primarily with AChE1 more than with AChE2. Therefore, we select the *ACE1* gene for silencing strategies in the context of ongoing debates regarding which protein performs a greater function in nerve transmission.

### 3.2. Design of RNAi and Off-Target Analysis

**Figure 1:**
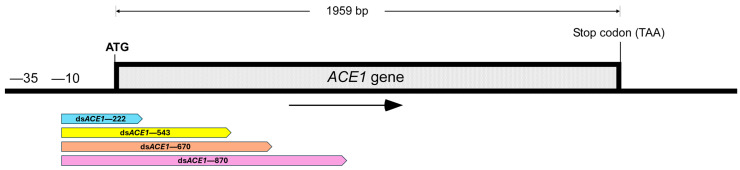
RNA sequences of varying lengths designed for the silencing of the *ACE1* gene (GenBank: JF343436) in *L. decemlineata*. All sequences target the 5′ region of the *ACE1* gene start from position −10 and exhibit an approximate GC content of 60%.

**Figure 2:**
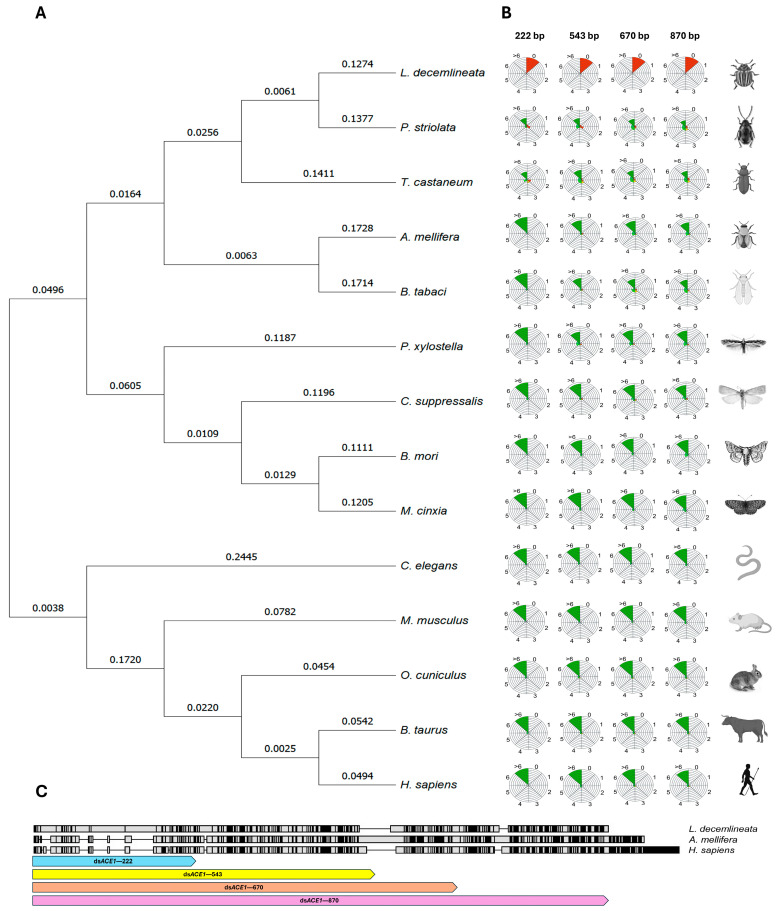
(**A**) Phylogenetic tree of acetylcholinesterase based on nucleic acid sequences from *L. decemlineata*, *P. striolata*, *T. castaneum*, *A. mellifera*, *B. tabaci*, *P. xylostella*, *C. suppressalis*, *B. mori*, *M. cinxia*, *C. elegans*, *M. musculus*, *O. cuniculus*, *B. taurus*, and *H. sapiens*, constructed using the Neighbor-Joining method with Molecular Evolutionary Genetics Analysis (MEGA) 11.0.10 software. (**B**) Theoretical potency analysis of each species was calculated using the QUT *Nicotiana benthamiana* RNA Target Tester platform. Circles indicate the theoretical effectiveness of siRNAs produced using each ds*ACE1* sequence designed for *L. decemlineata*. Twenty-one nucleotide siRNA sequences with 0, 1, or 2 mismatches are expected to target gene cleavage (red), while those with >3 mismatches are expected to have no significant RNAi effect (green). Sequences shown in orange are classified as potentially having an RNAi effect. (**C**) Alignment of acetylcholinesterase coding sequences from *H. sapiens*, *A. mellifera*, and *L. decemlineata*. Conserved residues are marked in gray and black boxes. The lower boxes indicate the target region of the ds*ACE1* sequences.

To investigate the effect of different lengths of RNAi on the *ACE1* gene, the *ACE1* sequence (JF343436) was used as a template. Four fragment lengths were designed starting at nucleotide −10: 222 bp (−10 to +212), 543 bp (−10 to +533), 670 bp (−10 to +660), and 870 bp (−10 to +860) (Figure 1). To assess the silencing potential of these four sequences, a bioinformatic analysis was performed using the RNA Target Tester, which generated all possible 21 bp oligomers. The analysis results indicate a high RNA interference potential across all fragments: the 222 bp fragment produced 192 unique siRNAs with a potency score of 95%, the 543 bp fragment generated 513 unique siRNAs with a potency score of 98.1%, the 670 bp fragment produced 640 unique siRNAs with a potency score of 98.5%, and the 870 bp fragment generated 840 unique siRNAs with a potency score of 98.8% (Figure 2).

At the same time, an off-target analysis was conducted to evaluate possible interference with acetylcholinesterase sequences from organisms such as *P. striolata, T. castaneum, A. mellifera*, *B. tabaci*, *P. xylostella*, *C. suppressalis*, *B. mori*, *M. cinxia*, *C. elegans*, *M. musculus*, *O. cuniculus*, *B. taurus*, and *H. sapiens*. The analysis confirmed that all four designed sequences exhibited more than three mismatches per 21-mer, indicating minimal off-target effects and ensuring specificity, thereby minimizing the risk of unintended silencing (Figure 2B,C). Based on this, the four sequences were provided with *Swa*I and *Avr*II restriction sites at the 5′ and 3′ ends, respectively. The final sequences were synthesized using GenScript Inc. (Piscataway, NJ, USA) and cloned into the pBJC4 vector (PQ589839) to form the pBJC5 (*ACE1*—222 bp), pBJC6 (*ACE1*—543 bp), pBJC7 (*ACE1*—670 bp), and pBJC8 (*ACE1*—870 bp) vectors, respectively. The vectors were deposited in GenBank with the accession numbers PQ615931, PQ589842, PQ589841, and PQ589840, respectively.

### 3.3. In Vitro Synthesis of dsRNA Targeting the ACE1 Sequence

**Figure 3:**
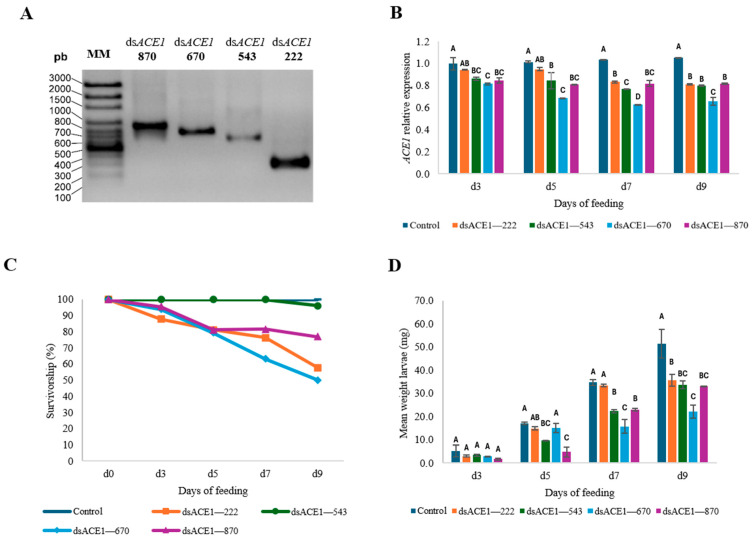
Synthesis and feeding assays of ds*ACE1* in *Leptinotarsa decemlineata*. (**A**) In vitro synthesis of ds*ACE1* transcripts of different lengths (222 bp, 543 bp, 670 bp, and 870 bp). (**B**) Relative expression levels of *ACE1* in *L. decemlineata* larvae. (**C**) Survival percentage and (**D**) average larval weight after 3, 5, 7, and 9 days of treatment with ds*ACE1*. Data are presented as means ± SD. Letters above each bar indicate significant differences between groups, determined using one-way ANOVA followed by Tukey’s test in Minitab^®^ 20.3.

Larval feeding assays were conducted using dsRNA targeting *ACE1*. The vectors pBJC5, pBJC6, pBJC7, and pBJC8, which contain the fragments encoding the *ACE1* gene for acetylcholinesterase, were used as templates for in vitro dsRNA synthesis. The in vitro synthesis yielded fragments of approximately 222 bp, 543 bp, 670 bp, and 870 bp, corresponding to the expected sizes of the designed sequences (Figure 3A).

### 3.4. Effects of Larval Feeding with dsRNA Targeting the ACE1 Gene

Larvae of the second instar from *L. decemlineata* were selected for insecticidal bioassays due to their higher susceptibility to dsRNA compared to third-instar larvae [79]. In the bioassays, larvae fed on leaves treated with dsRNA targeting *ACE1* (ds*ACE1*) (4 ng cm^−2^) at lengths of 222 bp, 543 bp, 670 bp, and 870 bp exhibited a decrease in transcripts. On day 3, transcript levels in larvae treated with ds*ACE1*—543, ds*ACE1*—670, and ds*ACE1*—870 were significantly reduced compared to the control, although differences among these treatments were not significant. In contrast, ds*ACE1*—222 did not significantly differ from the control. By days 5, 7, and 9, all ds*ACE1* treatments significantly reduced transcript levels relative to the control, with ds*ACE1*—670 producing the most pronounced reduction (Figure 3B).

Larvae that consumed ds*ACE1* showed higher mortality rates than the control throughout the experiment (Figure 3C). Notably, while ds*ACE1*—222 showed a greater reduction in survival compared to other lengths on day 3, larvae treated with ds*ACE1*—670 had the lowest survival rates overall by the end of the bioassay. Interestingly, larvae treated with ds*ACE1*—543 displayed survival rates similar to the control.

Regarding weight gain, no significant differences were observed on day 3 among any of the treatments. By day 5, however, larvae fed with ds*ACE1*—543 and ds*ACE1*—870 exhibited significant weight reductions compared to the control.

On day 7, larvae treated with ds*ACE1*—222 showed no significant differences in weight gain relative to the control. In contrast, larvae exposed to ds*ACE1*—543 and ds*ACE1*—870 showed notable weight loss. Remarkably, larvae treated with ds*ACE1*—670 exhibited the most significant weight reduction during this period (Figure 3D).

**Figure 4:**
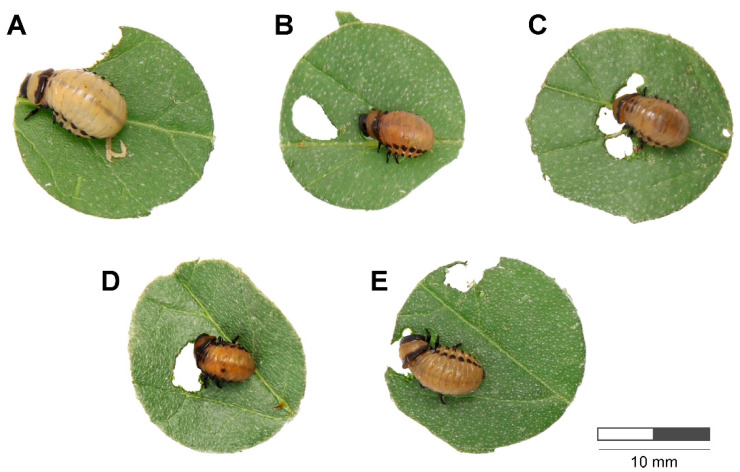
Phenotype of *L. decemlineata* larvae fed with silverleaf nightshade leaves painted with ds*ACE1* (4 ng cm^−2^) on the ninth day of treatment. (**A**) Larvae fed with leaves without ds*ACE1* (control), showing the typical phenotype. (**B**) Larvae fed with leaves painted with ds*ACE1*—222. (**C**) Larvae fed with leaves painted with ds*ACE1*—543. (**D**) Larvae fed with leaves painted with ds*ACE1*—670. (**E**) Larvae fed with leaves painted with ds*ACE1*—870.

The trend in weight reduction persisted through day 9. Larvae in the ds*ACE1*—222 treatment group displayed stagnation in weight gain, with no significant differences compared to the ds*ACE1*—543 and ds*ACE1*—870 groups. However, the ds*ACE1*—670 treatment continued to promote the most substantial weight loss among all treatments (Figure 3 and Figure 4).

The arrest in weight gain observed on days 7 and 9 correlated with the reduction in *ACE1* transcript levels. However, this correlation was not consistent across all time points, particularly on days 3 and 5, suggesting that additional factors beyond dsRNA length may contribute to the observed phenotypic effects.

To investigate this further, we analyzed whether differences in dsRNA conformation and stability could explain the variations in silencing efficiency. Since ds*ACE1* gene silencing correlated with dsRNA length—except for ds*ACE1*—870—and considering that all sequences have a similar GC content (~60%), we examined secondary structure formation and stability using RNAcofold. The analysis revealed a progressive increase in structural stability and diversity with longer sequences. The ds*ACE1*—222 sequence had a ΔG (minimum free energy, MFE) of −517.70 kcal/mol and a frequency of 31.35% in the thermodynamic ensemble, indicating a higher tendency to adopt a single, stable conformation. In contrast, ds*ACE1*—543 exhibited a ΔG of −1257.80 kcal/mol and a frequency of 5.67%, indicating a greater tendency to form multiple conformations. Both stability and conformational diversity increased with dsRNA length. For instance, ds*ACE1*—670 showed a ΔG of −1545.20 kcal/mol with a frequency of 5.42%, while ds*ACE1*—870 had a ΔG of −2004.20 kcal/mol and a frequency of 2.01%. Although longer sequences, particularly ds*ACE1*—870, exhibit greater stability, they may also adopt a broader range of conformations, potentially compromising their gene-silencing efficiency.

## 4. Discussion

Pest control is essential for food security, as approximately 40% of agricultural production is lost before harvest due to insect damage, with an additional 20% lost post-harvest [80]. The Colorado Potato Beetle is among the most destructive pests, earning the label of “super pest” due to its defoliation capacity and rapid development of insecticide resistance. Although the exact mechanisms of CPB’s rapid adaptation to new toxins are not fully understood [53,62,81], research suggests that resistance may be due to multiple factors, including target site insensitivity, reduced penetration, and a complex network of xenobiotic pathways [62,82,83]. This resistance is further exacerbated by the repeated use of the same insecticide classes without rotating active ingredients [43]. The particular characteristics of CPB, along with poor management practices [84], have led to severe economic losses.

Given the challenges posed by insect resistance, various strategies have been explored to control *L. decemlineata*, including genetic approaches like RNAi that have stood out due to their low impact on the ecosystem and their high specificity [68,85]. In fact, Calantha^TM^ (active ingredient Ledprona), an RNAi-based bioinsecticide, is already commercially available and has shown effectiveness in controlling *L. decemlineata* at low doses [43,86]. However, recent studies indicate that its efficacy may vary across different geographic populations [87], underscoring the need for further research to identify the most effective strategies for controlling the beetle.

To date, more than 26 genes have been targeted for the control of CPB, starting with *β-Actin*, *Sec23*, *ATPase, COPβ*, and more recently, *Mesh*, *Ran*, *Tai*, *EcR*, *HR4*, and *CncC* [22,24,48,58,61,62,63,64]. Nevertheless, while gene silencing has generally resulted in growth retardation and, in some cases, mortality in larvae, there remains a need to identify target genes that maximize RNA interference efficiency. These genes should influence key biological processes, such as development, reproduction, stress resistance, or metabolism, with minimal impact on the ecosystem. Such refinements are crucial for improving RNAi technology.

In this context, this study focused on acetylcholinesterase, an enzyme responsible for terminating neuronal transmission by breaking down acetylcholine at synapses [88], as its inhibition can disrupt key nervous system functions in pests [28,72,73]. We found that silencing the *ACE1* gene, which encodes the AChE1 isoform, significantly reduces transcription levels and impairs larval growth when treated with various dsRNA lengths.

Acetylcholinesterase has two isoforms in most insects, AChE1 and AChE2, which are encoded by the *ACE1* and *ACE2* genes [89]. While AChE1 serves as the primary enzyme for terminating synaptic transmission in many species [86,87,90,91], in insects such as *A. mellifera*, *Musca domestica,* and *Drosophila melanogaster*, the activity primarily depends on AChE2 [89,92,93]. Interestingly, in *D. melanogaster*, it has been proposed that *ACE2* can generate multiple isoforms of AChE2 through alternative splicing [89].

In *L. decemlineata*, it was also proposed that AChE1 is the primary enzyme responsible for terminating neuronal transmission, leaving AChE2 with a secondary role [77]. However, recent studies suggest that AChE2 may actually serve as the main catalytic enzyme, with AChE1 acting more as a bioscavenger [94]. These differing findings continue the debate regarding which gene predominantly encodes the critical enzyme for inhibiting neurotransmission in *L. decemlineata*. Supported by in silico analysis of sequence, structural, and interaction data, we determined that *ACE1* is the most suitable gene to silence the acetylcholinesterase in the synapsis.

Although gene targeting is a crucial step in RNAi design, our primary goal was to evaluate the effect of different lengths of dsRNA targeting the *ACE1* gene. Therefore, we designed four vectors carrying different lengths of dsRNA targeting the *ACE1* gene of *L. decemlineata* (ds*ACE1*—222, ds*ACE1*—543, ds*ACE1*—670, and ds*ACE1*—870).

Our results demonstrated that the ds*ACE1*—670 bp length induced the most significant silencing of the *ACE1* gene, with transcript levels decreasing by approximately 40% on the seventh day compared to the control and maintaining a reduction of 37.8% by the ninth day. The ds*ACE1*—543 bp length also exhibited a moderate silencing effect, with a reduction of about 25.7% on day seven, followed by a slight rebound to 24% by day nine. In contrast, although ds*ACE1*—222 and ds*ACE1*—870 treatments resulted in significant transcript reductions compared to the control, no significant difference was observed between them, with only a marginal difference of 1.8%. These findings suggest that both shorter (222 bp) and longer (870 bp) dsRNA lengths are less effective in silencing the *ACE1* gene compared to intermediate lengths.

These results suggest that an optimal dsRNA length (~670 bp) is crucial for effective gene silencing of the *ACE1* gene in *L. decemlineata*, potentially due to better processing and incorporation into the RNAi machinery.

These findings differ from previous reports on dsRNA silencing efficiency in *L. decemlineata*. For instance, silencing the *β-actin* gene using dsRNA of varying lengths (200 bp, 297 bp, 400 bp, and 700 bp) showed little difference in transcript decline and mortality [68]. Although dsRNA length is widely regarded as a critical factor for effective silencing [95], studies across different laboratories have produced varied results, suggesting that the optimal length may depend on multiple factors, including the target gene and the insect species (Table 1).

In line with this, several studies have used different dsRNA lengths with varied outcomes. For example, silencing the *snf7* gene in *D. virgifera virgifera* was achieved with a 240 bp dsRNA [96], while a 220 bp dsRNA successfully silenced the *β-actin* gene in *B. tabaci* [69]. Similarly, the *ACE* gene was silenced effectively with a 189 bp dsRNA in *H. armigera* [23], and a 275 bp dsRNA targeting the *vATPase C* gene caused significant mortality in *D. virgifera virgifera* [97]. Other studies also demonstrate that dsRNA lengths ranging from 364 bp to 520 bp can silence genes with varying levels of efficacy in different species [46,70,71].

The variability in results highlights that dsRNA length alone is not the sole determinant of silencing success. The specific gene targeted also plays a critical role in determining the efficacy of RNAi treatments. For instance, while dsRNA targeting the *vATPase E* and *vATPase B* genes decreased transcript levels by 93%, silencing of other genes, such as *actin*, *sec23*, and *COPβ*, resulted in lower transcript reductions of 61%, 83%, and 79%, respectively [22].

In our study, silencing the *ACE1* gene with dsRNAs of varying lengths resulted in different levels of efficacy. The ds*ACE1*—670 treatment exhibited the highest mortality, with only 63% of larvae surviving compared to the control. This was followed by ds*ACE1*—222, which resulted in a survival rate of 76%, whereas ds*ACE1*—543 and ds*ACE1*—870 treatments showed no significant effects on survival. By day 9, cumulative mortality was still highest in the ds*ACE1*—670 treatment (50%), while ds*ACE1*—222 showed a survival rate of 58%. These findings highlight the importance of dsRNA length, with an optimal length (~670 bp) being critical for effective silencing of the *ACE1* gene in *L. decemlineata*.

Our results also indicate that shorter lengths, such as 222 bp, can still show moderate effectiveness in silencing gene expression, but their impact on mortality may be less pronounced in the short term. The results for ds*ACE1*—543 and ds*ACE1*—870, which did not exhibit significant mortality effects, highlight the potential complexity of dsRNA processing and stability.

Different mortality and growth inhibition levels have also been obtained with the silencing of different genes and lengths in *L. decemlineata*. For instance, silencing the *Ran* gene resulted in complete larval growth inhibition and 100% mortality within just three days [48], while targeting the *Tai* gene achieved around 20% larval mortality and an 80% pupation failure rate [64]. Similarly, *SAHase* gene silencing induced 42% mortality after 12 days of continuous dsRNA ingestion [47]. Furthermore, there is not always a correlation between transcript levels, weight gain, and mortality [59,60]. These observations indicate that RNAi outcomes, including timing and severity of mortality, vary significantly depending on the gene targeted (Table 1). In this regard, not all essential genes are equally suitable for silencing, as they do not all exhibit the same level of insecticidal activity [95]. Insect control is likely to be more efficient when dsRNAs are directed toward transcription factors that regulate essential genes [62], particularly those with crucial functions in the early stages of insect development.

In RNAi design, minimizing secondary structure formation has been suggested to enhance gene silencing, as highly stable secondary structures may hinder dsRNA interaction with Dicer. However, this approach may be more suitable when dsRNA is produced endogenously within cells (e.g., in transgenic systems), where rapid degradation by environmental factors is less of a concern.

In our study, ds*ACE1*—222, with a relatively high MFE (ΔG = −517.70 kcal/mol) and low structural diversity, exhibited fewer secondary structures. While this could facilitate processing by Dicer, the reduced structural diversity might also increase its susceptibility to degradation when administered externally, where it is exposed to various environmental factors [98]. On the other hand, ds*ACE1*—543 and ds*ACE1*—670, with lower MFE values (ΔG of −1257.80 and −1545.20 kcal/mol, respectively), are more likely to form stable structures that may offer greater resistance to degradation in extracellular environments, such as the insect’s digestive tract. This enhanced structural stability might allow for some of the dsRNAs to persist for longer, potentially reaching target cells in certain tissues [99,100,101,102]. In contrast, ds*ACE1*—870, although highly stable (ΔG of −2004.20 kcal/mol), exhibits significant structural diversity with a frequency of 2.01% in the thermodynamic ensemble, which may indicate the presence of multiple rigid conformations that could hinder efficient unfolding and subsequent interaction with Dicer [103,104], potentially reducing its efficacy in gene silencing.

We hypothesize that an optimal balance between structural stability and flexibility is crucial for effective gene silencing using in vitro synthesized dsRNA administered via diet. While a certain degree of stability is necessary to protect dsRNA from degradation before absorption and processing by Dicer, excessive stability could impede Dicer’s ability to process the dsRNA efficiently. Sequences with moderate structural stability and balanced diversity may offer the best performance, protecting against degradation while retaining the flexibility needed for Dicer processing and effective gene silencing. However, given the complexity of these interactions, further studies are essential to establish a clear relationship between dsRNA structure and silencing efficacy.

The variable results obtained with different dsRNA lengths may be influenced by several factors, such as the specific region of the mRNA targeted. For example, silencing efficiency can vary even when equal-length dsRNA targets different positions of the mRNA [97]. While some studies suggest that targeting dsRNA to the 3′ regions of mRNA improves silencing, others have shown enhanced silencing when the 5′ regions are targeted [24,67,97].

Our results support the idea that dsRNA length is a critical factor in silencing efficiency, but it must be considered alongside other important variables, such as the targeted gene, the specific insect species, the delivery method, and the processing of dsRNA within the organism [25,68,71,97,105,106,107,108]. Additionally, strategies that modulate the GC content to enhance structural stability, along with the combination of dsRNAs of varying lengths targeting multiple mRNA regions, may further increase the likelihood of effective gene knockdown and help circumvent sequence-specific resistance mechanisms. However, while these parameters are crucial, they are not yet fully understood. Therefore, further investigation should focus on optimizing dsRNA length while integrating these additional factors to improve the consistency and effectiveness of RNAi-based pest management strategies.

## 5. Conclusions

Our study demonstrates that silencing the *ACE1* gene in *L. decemlineata* is most efficient using dsRNA of 670 bp, compared to both shorter and longer lengths. These results reinforce the idea that dsRNA length is critical for silencing efficiency. However, it is important to note that length should not be evaluated in isolation, but alongside other factors such as the target gene, the specific sequence, and the amount of dsRNA administered [23]. Despite the challenges associated with designing effective RNA interference, this technology shows significant potential as a tool for pest control, particularly given the increasing resistance observed in various organisms to conventional control methods. In fact, to date, the APRD has documented more than 19,500 resistance cases across 632 pest species. Consequently, the successful application of RNAi in agriculture will depend not only on optimizing dsRNA length, but also on improving RNAi delivery strategies and developing methods to delay resistance development in pest populations [105,106] while integrating it with other pest control strategies.

## Figures and Tables

**Table 1:** RNAi-mediated gene silencing in *Leptinotarsa decemlineata* and its phenotypic effects.

Gene	Length (bp)	Production	Amount	mRNA Reduction	Effects	Reference
*Sec23*	1506	In vitro synthesis	50 µg	83%	Mortality and reduced weight gain.	[22]
*ATPase E*	469			93%		
*ATPase B*	530			93%		
*COPβ*	228			79%		
*β-Actin*	279	Modified plants	50 µg	—	Mortality and reduced growth.	[22,24]
	201		—	61%		
*FTZ-F1*	319	Bacterial production	0.5 µg∙µL^−1^	70.2%	Failure of ecdysis and mortality.	[44]
*Prohibitin-1*	350	In vitro synthesis	10 µg	90%	Mortality and reduced growth.	[45]
*Shd*	438	Bacterial production	—	46.8%	Mortality and reduced weight.	[46]
*SAHase*	521	Bacterial production	—	84%	Decreased larval weight, failed pre-pupation, reduced larval development time, and mortality.	[47]
*Ran*	361	Bacterial production	0.5 µg∙mL^−1^	~90%	Weight reduction and mortality.	[48]
*NAT1*	357	Bacterial production	0.5 µg∙mL^−1^	~90%	Delayed larval growth and impaired pupation.	[49]
*HR3*	141	Bacterial production	0.05 µg∙µL^−1^	~70%	Pupation failure, cuticle defects, and mortality.	[50]
*JHEH*	284–316	Bacterial production	0.05 µg∙µL^−1^	94.5%	Slightly reduced larval weight and delayed larval development with impaired adult emergence.	[51]
*alt 1*	398	Bacterial production	0.5 µg∙µL^−1^	79.5%	Flight impairment and mortality.	[52]
*alt 2*	392			71.1%		
*p5cdh1*	490	Bacterial production	0.5 µg∙µL^−1^	~60%	Reduced ATP content, flight impairment, and mortality.	[53]
*p5cdh2*	473			~70%		
*UAP1*	367	Bacterial production	0.5 µg∙mL^−1^	~70%	Impaired molting and mortality.	[54]
*UAP2*	331					
*ChSA* (*a*,*b*)	~300	Bacterial production	0.5 µg∙mL^−1^	~80%	Failure of ecdysis, emergence, decreased chitin content, and delayed larval growth.	[55]
*ChSB*	~500					
*TPS*	325/500	Bacterial production	0.5 µg∙µL^−1^	-	Mortality, with surviving larvae showing greater body mass, glycogen, lipids, and proline but reduced chitin content.	[56]
*TRE*	426/414					
*E75A*, *B* and *C*	~300	Bacterial production	0.5 µg∙µL^−1^	~80%	Average pupal weight with pupation arrest.	[57]
*Mesh*	417	In vitro synthesis	0.5 µg∙µL^−1^	71%	Mortality and lower adult emergence rate.	[58]
*JHAMT1*	261	Bacterial production	0.05 µg∙µL^−1^	98.3%	Death of larvae, weight loss, shortened larval development period, and impaired pupation.	[59]
*JHAMT2*	332			93.2%		
*ILP1*	201	Bacterial production	0.05 µg∙µL^−1^	80%	Impaired molting.	[60]
*ILP2*	179					
*HR4*	337	Bacterial production	0.5 µg∙mL^−1^	80%	Failure in pupation and mortality.	[61]
*CncC*	—	In vitro synthesis	5 µg∙µL^−1^	~80%	Decreased expression of ABC transporters, which reduces resistance to imidacloprid.	[62]
*EcR*	445 bp	Transgenic plants	—	~55%	Mortality and reduction in larval weight.	[63]
*Tai*	—	Bacterial production	0.5 µg∙mL^−1^	80%	Pupation failure, cuticle defects, and mortality.	[64]

**Table 2:** Primers used for in vitro synthesis dsRNA.

Length (bp)	Sequence (5′—3′)
	First amplicon
222	Fw—5′TAATACGACTCACTATAGGGGGAGCGACGATGACAACAACG3′
	Rv—5′TCTCTCCTGGTGAAGTCGCGTT3′
543	Fw—5′TAATACGACTCACTATAGGGGGAGCGACGATGACAACAACG3′
	Rv—5′CTCGGCTTCGGCACCACCACGT3′
670	Fw—5′TAATACGACTCACTATAGGGGGAGCGACGATGACAACAACG3′
	Rv —5′CTGCATCGACACCAGAATGATA3′
870	Fw—5′TAATACGACTCACTATAGGGGGAGCGACGATGACAACAACG3′
	Rv—5′GATAGCGGCGAGAAGAGGTGAA3′
	Second amplicon
222	Fw—5′GGGAGCGACGATGACAACAACG3′
	Rv—5′TAATACGACTCACTATAGGTCTCTCCTGGTGAAGTCGCGTT3′
543	Fw—5′GGGAGCGACGATGACAACAACG3′
	Rv—5′TAATACGACTCACTATAGGCTCGGCTTCGGCACCACCACGT3′
670	Fw—5′GGGAGCGACGATGACAACAACG3′
	Rv—5′TAATACGACTCACTATAGGCTGCATCGACACCAGAATGATA3′
870	Fw—5′GGGAGCGACGATGACAACAACG3′
	Rv—5′TAATACGACTCACTATAGGGATAGCGGCGAGAAGAGGTGAA3′

## Data Availability

The original contributions presented in this study are included in the article. Further inquiries can be directed to the corresponding author.
